# Effects of *MrwetA* on Sexual Reproduction and Secondary Metabolism of *Monascus ruber* M7 Based on Transcriptome Analysis

**DOI:** 10.3390/jof10050338

**Published:** 2024-05-08

**Authors:** Yuyun Huang, Lili Jia, Fusheng Chen

**Affiliations:** 1 National Key Laboratory of Agricultural Microbiology, Huazhong Agricultural University, Wuhan 430070, China; 2 Hubei International Scientific and Technological Cooperation Base of Traditional Fermented Foods, Huazhong Agricultural University, Wuhan 430070, China; 3 College of Food Science and Technology, Huazhong Agricultural University, Wuhan 430070, China

**Keywords:** *Monascus ruber*, sexual reproduction, ascospore, secondary metabolism

## Abstract

*wetA*, one of the conidiation center regulatory genes in many filamentous fungi, plays an important role in promoting asexual spores (conidia) maturation. Our recent research has found that knocking out or overexpressing *MrwetA* (a homolog of *wetA*) in *Monascus ruber* M7 does not affect the development of its asexual spores like other fungi, but both repress the development of its sexual spores (ascospores). However, the mechanism remains unclear. In this study, the function of *MrwetA* on sexual reproduction and secondary metabolism in *M. ruber* M7 was confirmed by a complementary experiment. Moreover, the regulatory roles of *MrwetA* in modulating the expression of genes involved in sexual reproduction, meiosis, and biosynthesis of *Monascus* pigment and citrinin were analyzed based on the transcriptional data. These results not only contribute to clarifying the regulation of the reproduction and secondary metabolism of *Monascus* spp., but also to enriching the regulation molecular mechanism of reproduction in filamentous fungi.

## 1. Introduction

*Monascus* spp., a sort of filamentous fungi used to produce fermented foods and medicines, possess a history of application for nearly 2000 years in China and other Asian countries [1,2]. *Monascus* spp. are highly valued due to their ability to produce a diverse range of beneficial secondary metabolites, such as *Monascus* pigments (MPs) used as food colorants [3,4], and monacolin K (MK), a functional ingredient for lowering blood pressure [5]. Although some *Monascus* strains may also produce citrinin (CIT) [6], the CIT issue in *Monascus*-relative products has been well controlled through the unremitting efforts of scientists [7,8,9,10]. Over the past decades, numerous studies have investigated the biosynthesis of MPs, CIT, and MK in *Monascus* spp. And their gene clusters and biosynthesis pathways have been well-established [1,11,12,13]. However, due to the long fermentation time of *Monascus*-relative products, their production cost is often high and they are prone to contamination by miscellaneous microorganisms, resulting in food safety issues. Therefore, how to promote the growth of *Monascus* strains, and shorten their fermentation time is a major challenge at present [14]. 

*Monascus* spp. can produce asexual (conidia) and sexual (ascospores) spores, and both spores are very important for *Monascus* reproduction and growth [15]. The mechanisms by which the *Monascus* genus produces asexual and sexual spores were previously believed to be similar to the *Aspergillus* genus due to their close phylogenesis [16,17,18]. However, our recent research has found that in the *Monascus* genomes, there is no homologous of *abaA*, one of the core asexual development regulatory genes [19]. Additionally, our findings indicate that *MrwetA*, homologous of another central asexual development regulatory gene *wetA*, is not involved in regulating asexual spore production but rather plays a role in sexual reproduction in *M*. *ruber* M7 [20]. Former research revealed that *wetA* played a conserved role in conidial maturation and cell wall synthesis in *Aspergillus* spp., including *A. nidulans*, *A. fumigatus*, *A. oryzae*, and *A. flavus* [21,22,23,24,25,26]. Furthermore, *wetA* has been widely reported to function in forming asexual spores not only restricted to *Aspergillus* spp. [27,28], but also in a wide range of Ascomycota strains, including *Penicillium chrysogenum*, *P. digitatum*, *Metarhizium robertsii*, and *Beauveria bassiana* [29,30,31,32,33]. Although a recent study revealed that deletion of *wetA* caused 52%, 57%, and 43% of all genes differentially expressed in *A. nidulans*, *A. fumigatus*, and *A. flavus* [34], there is no direct evidence that *wetA* may participate in the sexual cycle. Our recent research first reported that *MrwetA* could regulate its sexual development, with both the deletant and overexpression mutants [20]. However, the mechanism remains unclear.

In this study, at first, *MrwetA* was complemented to *MrwetA* knockout mutant and its phenotypes were compared with those of *M. ruber* M7 and *MrwetA* knockout mutant. Furthermore, the differentially expressed genes between *MrwetA* knockout and *M. ruber* M7 were systematically analyzed to reveal the underlying mechanism. The results revealed that *MrwetA* did affect the sexual development and secondary metabolites by regulating the expression levels of genes involved in sexual reproduction, meiosis, and the biosynthesis of MPs and CIT in *M. ruber* M7. These findings will help to clarify the regulatory mechanisms of the reproduction and secondary metabolism of *Monascus* spp.

## 2. Materials and Methods

### 2.1. Strains and Culture Conditions

*M. ruber* M7 (CCAM 070120, Culture Collection of State Key Laboratory of Agricultural Microbiology, Wuhan, China), which is used as the wild strain (WT) and can produce MPs and CIT [35]. Δ*MrwetA*, is a knockout strain with *hph* as resistance marker [20], Both strains are described in Table 1.

Potato dextrose agar (PDA), malt extract agar (MA), Czapek yeast extract agar (CYA), and 25% glycerol nitrate agar (G25N) were utilized for morphological characteristics. PDA medium was used for the analyses of MPs and CIT production. Inducing medium (IM) was used to activate *Agrobacterium tumefaciens* before co-culture, and coculture-inducing medium (Co-IM) was used to generate mutants for the co-culture process. To select mutants, PDA supplied with 15 μg/mL G418 sulfate (Sigma-Aldrich, Shanghai, China) was used to isolate neomycin-resistant transformants. All strains were maintained on PDA slants at 28 °C.

### 2.2. Construction of the MrwetA Complemented Strain

In our recent research, the mutants of knocking out and overexpressing *MrwetA* were prepared [20]. In this study, the *MrwetA* complementation strain was constructed, according to the strategy of homologous recombination [36,37]. The genomic DNA of M7 was used to amplify the complete *MrwetA* gene sequence (1659 bp), 5′ flanking region (1052 bp), and 3′ flanking region (915 bp). *neo* (neomycin phosphotransferase resistance gene, 1221 bp) as the selected maker was amplified from plasmid pKNI. *hph* (hygromycin-B resistance marker gene, 2137 bp) as a maker for screening Δ*MrwetA*. The relevant primer pairs are shown in Table S1. 

The construction steps of the complementation strain are as follows. Firstly, these cloned DNA fragments were ligated to plasmid pCAMBIA3300 (digested with *Sal* I and *Hin*d III) by using pEASY^®^-Basic Seamless Cloning and Assembly Kit (TransGen Biotech, Beijing, China) to form plasmid pCCMrwetA. The construction procedure is shown in Figure S1. Secondly, the plasmid pCCMrwetA was transformed into *A. tumefaciens* EHA105 using a freeze-thaw method. Finally, the *A. tumefaciens* EHA105 clones containing pCCMrwetA were incubated for transformation with the ∆*MrwetA* strain to generate the *MrwetA* complementation strains (∆*MrwetA*::*MrwetA*).

### 2.3. Observations of Sexual Reproduction

Referred to Jia et al. [20], WT, Δ*MrwetA*, and Δ*MrwetA*::*MrwetA* strains were cultured for 9 d at 28 °C to observe microscopic morphologies on PDA, CYA, G25N, and MA. To measure the number of cleistothecia and ascospores, freshly harvested spores (1 × 10^5^/mL) from each strain were inoculated in a PDA plate covered with sterile cellophane and incubated at 28 °C. Samples collected at every 4 d from the 3rd to the 11th d were scraped. The cleistothecia were counted using a five-point sampling method. Colonies with an area of 1 per cm^2^ were collected from each point, washed with 1 mL of sterile water, and counted under a microscope. And the number of ascospores was counted after breaking the cleistothecia.

### 2.4. Detection of Monascus Pigments and Citrinin

To detect the yield of MPs and CIT, 200 μL freshly harvested spores (1 × 10^5^/mL) from each strain were inoculated onto Pa DA plate covered with sterile cellophane and incubated at 28 °C for 11 d. Hyphae and media were respectively collected at every 2 d from the 3rd to the 11th d, and freeze-dried. Then, the freeze-dried hyphae (20 mg) and media (20 mg) were separately ground into powder. Then, 1.5 mL of 80% (*v*/*v*) methanol (chromatographic grade) was added and mixed to extract under ultrasonic (KQ-250B, Kunshan, China) for 30 min at 30 °C. After centrifugation at 12,000× *g* for 10 min, the supernatants were used for measuring MPs and CIT. 

The supernatant solution was diluted to an appropriate concentration with 80% methanol, and 80% (*v*/*v*) methanol was used as the control (CK). The yellow MPs, orange MPs, and red MPs were detected at 380 nm, 470 nm, and 520 nm using a UV-visible spectro-photometer (UV-1700, Shimadzu, Tokyo, Japan). Then, absorption values were multiplied by the dilution factor to obtain the yields of yellow, orange, and red MPs. In addition, the CIT content was determined by ultra-high performance liquid chromatography with the fluorescence detector (UPLC-FLR) following our previous paper [38].

### 2.5. RNA Extraction and RNA Sequencing

WT and Δ*MrwetA* were incubated at 28 °C on a cellophane-covered PDA plate. Two replicates were conducted for each strain. The fresh hyphae were obtained on the 3rd, 5th, and 7th d, respectively. The total RNA was extracted by using TransZol Up Plus RNA Kit (TransGen Biotech, Beijing, China). RNA sequencing was performed on DNBSEQ high-throughput sequencing platform (BGI, Wuhan, China, http://en.genomics.cn/ (accessed on 8 January 2022)). The isolation of RNA, construction of libraries, sequencing, data filtering, and quality control were performed according to the platform’s operation guides. The reference genome is the genome of *M. ruber* M7 sequenced by our lab [1].

Gene expression levels were calculated as RNA-Seq by Expectation-Maximization (RSEM) [39]. The normalized value of fragments per kilobase transcript fragment per million map readings (FPKM) was used as a parameter to compare expression levels between WT and Δ*MrwetA*. The differentially expressed genes (DEGs) were detected by DEseq software package (DEseq 2) [40]. DEGs were selected based on the standard of gene fold change (FC) ≥1.5 (|log_2_ FC| ≥ 0.5849) and Q value (adjusted *p*-value) ≤ 0.05. The Kyoto Encyclopedia of Genes and Genomes (KEGG, https://www.kegg.jp/ (accessed on 23 April 2022)) pathway is a database resource for understanding the high-level function and utility of biological systems, allowing analysis of the function of DEGs and the metabolic pathways involved. In addition, genes involved in fungal sexual reproduction and secondary metabolism were further analyzed to elucidate the role of *MrwetA* in sexual reproduction and secondary metabolism in *M. ruber* M7.

### 2.6. Statistical Analysis

*p* values were determined by using a one-way analysis of variance (ANOVA) test. A significance level of *p* < 0.05 was labeled with *, while *p* < 0.01 was denoted with **. Origin 2022 was utilized for graph plotting and statistical analysis. Error bars given in the figures represent the standard error.

## 3. Results

### 3.1. Construction of the MrwetA Complemented Strain

Following the successful transformation of the constructed and verified recombinant vector (Figure S2) into Δ*MrwetA*, colonies with resistance to G418 sulfate were selected and subsequent PCR was conducted (primers in Table S2) to screen the complemented strains. *MrwetA* knockout strains were constructed in the previous study, and *hph* was used as the resistance screening marker [20]. Putative target transformants were discerned by the amplification of *MrwetA*, *neo*, and *hph* fragments with lengths consistent with the expected sizes (Figure S3). In addition, PCR analysis confirmed the presence of *MrwetA* ORF and *neo* sequences as well as the absence of the hygromycin B sequence.

### 3.2. Reproduction Observations of M. ruber M 7 and Its MrwetA Modified Strains

**Figure 1:**
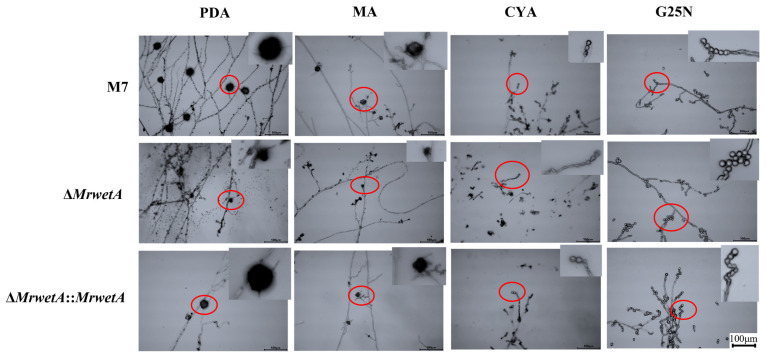
Microscopic morphology of WT, Δ*MrwetA*, and Δ*MrwetA*::*MrwetA* on PDA, MA, CYA, and G25N media (Size bar = 100 µm, 28 °C, 9 d). The red circle represents the magnified part.

Compared to *M. ruber* M7 (WT), Δ*MrwetA*, and Δ*MrwetA*::*MrwetA* exhibited no significant difference in conidial microscope morphologies across the four media (PDA, MA, CYA, and G25N), which are commonly used for morphological observation of *Monascus* spp. and cleistothecia could only be observed when cultivated on PDA and MA. As shown in Figure 1, the cleistothecia were similar in the WT and Δ*MrwetA*::*MrwetA*, whereas the sizes of cleistothecia were smaller in Δ*MrwetA* compared to them.

**Figure 2:**
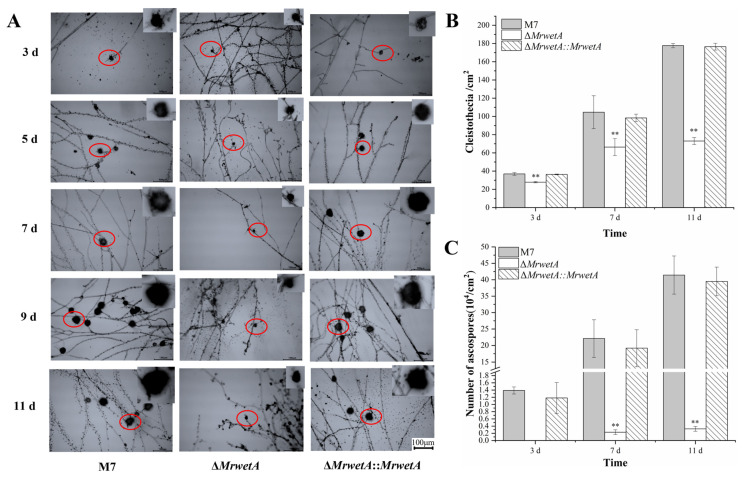
The cleistothecia and ascospores of WT, Δ*MrwetA*, and Δ*MrwetA*::*MrwetA.* (**A**) Microscopic morphological observation of the cleistothecia (Size bar = 100 µm, 28 °C, PDA). Columns indicate different strains, and rows indicate different time points. (**B**) Variation in the number of cleistothecia (28 °C, PDA). (**C**) Variation in the number of ascospores (28 °C, PDA). The red circle represents the magnified part. The symbol ‘**’ indicated an extremely significant difference (*p* < 0.01) compared with the WT.

The morphologies and yields of cleistothecia were analyzed from the 3rd d to the 11th d, to determine whether *MrwetA* delays or inhibits sexual development. The cleistothecia size in both WT and Δ*MrwetA*::*MrwetA* steadily increased from the 3rd to the 11th d. However, in Δ*MrwetA*, there was no significant change observed in cleistothecia size as time progressed, which remained smaller compared to that in the WT (Figure 2A). As shown in Figure 2B,C, the number of cleistothecia and ascospores was significantly reduced in Δ*MrwetA* compared to those in WT, whereas this defect could be restored in Δ*MrwetA*::*MrwetA*. Specifically, the number of cleistothecia in WT reached 36.93 ± 1.54/cm^2^, 104.67 ± 18.05/cm^2^, and 177.67 ± 2.38/cm^2^ on the 3rd, 7th, and 11th d, respectively, which was 1.33, 1.58, and 2.43 times higher than its yield in Δ*MrwetA*. Correspondingly, the WT ascospore numbers reached (2.21 ± 0.57) × 10^5^ (per cm^2^) and (4.14 ± 0.58) × 10^5^ (per cm^2^) on the 7th and 11th d, respectively, which were 96.22 and 129.50 times of that in Δ*MrwetA*.

### 3.3. MPs and CIT Analysis of WT and Its MrwetA Modified Strains

**Figure 3:**
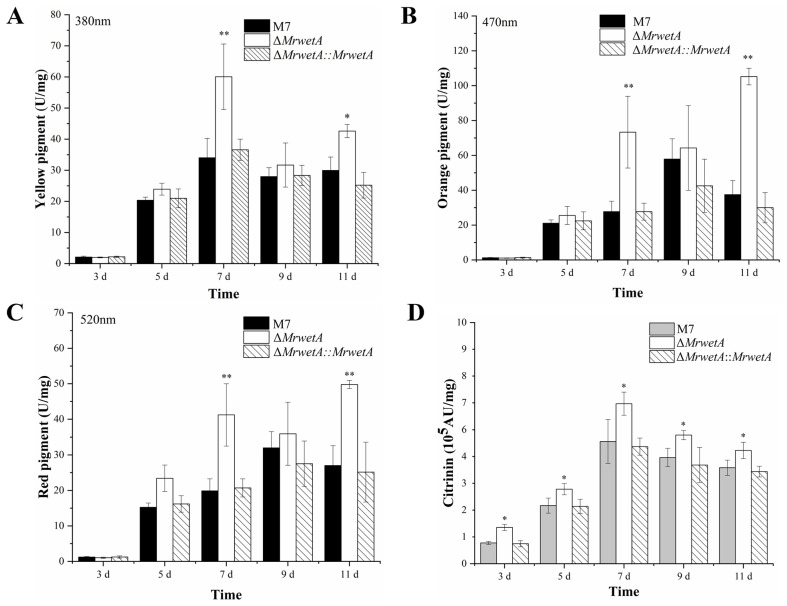
The production of *Monascus* pigments and citrinin in WT, Δ*MrwetA*, and Δ*MrwetA*::*MrwetA*. (**A**) The yield of yellow pigment (380 nm). (**B**) The yield of orange pigment (470 nm). (**C**) The yield of red pigment (520 nm). (**D**) The yield of citrinin. The symbol ‘*’ indicated a significant difference (*p* < 0.05) compared with the WT, while ‘**’ indicated an extremely significant difference (*p* < 0.01).

The effects of *MrwetA* deletion on secondary metabolites were investigated by assessing the production of MPs and CIT, as illustrated in Figure 3. Compared to the WT, Δ*MrwetA* exhibited no significant difference in yellow, orange, and red MP yields during the early stage (3 d–5 d), but showed an increase on the 7th and 11th d, while the MPs content of Δ*MrwetA*::*MrwetA* closely resembled that of WT across different time points (Figure 3A–C). In addition, the yield of CIT in Δ*MrwetA* was significantly increased at all time points compared to those in the WT, with no significant difference observed in Δ*MrwetA*::*MrwetA* (Figure 3D). The yield of total MPs and citrinin in the *MrwetA* overexpressed strain was higher on the 7th d compared to WT, similar to the Δ*MrwetA* [20]. In summary, the deletion of *MrwetA* resulted in enhanced MP production in the later period and CIT production in all observed time points.

### 3.4. The Transcriptomic Analysis of WT and ΔMrwetA

High-throughput sequencing technology was used to elucidate the mechanism of *MrwetA* in M7 on the transcription level. Based on the previous findings [20] and the results in this study, the deletion of *MrwetA* disturbed the sexual development and secondary metabolites, while complementation of *MrwetA* restored these disturbances, verifying the important role of *MrwetA* in the strain. As described by Yang et al. [41], when cultivated on PDA, M7 was at a logarithmic phase on the 3rd d, followed by stable growth from the 4th to the 7th d, and subsequently entering the decline phase after the 7th d. Consequently, samples were collected and analyzed on the 3rd, 5th, and 7th d in this study.

#### 3.4.1. Analysis of Differentially Expressed Genes

**Figure 4:**
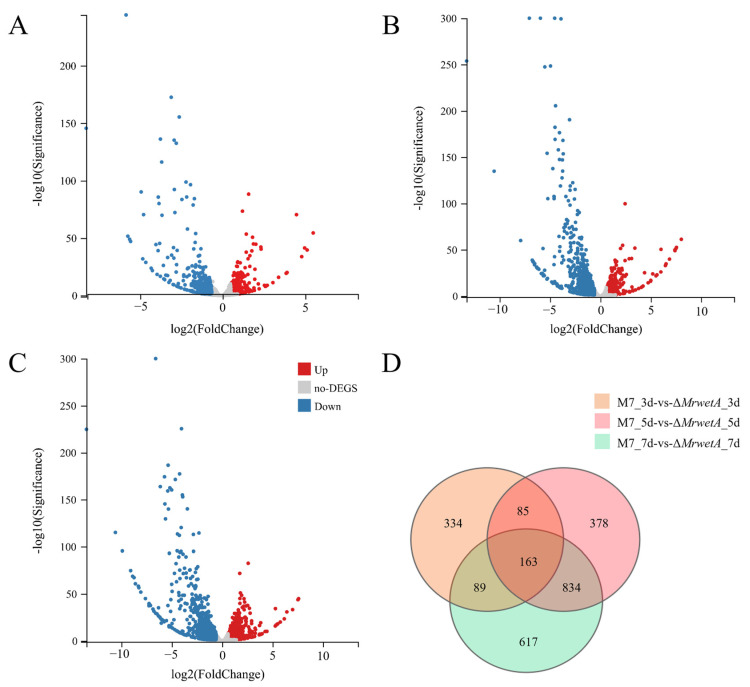
The number of DEGs. (**A**) Number of DEGs on the 3rd d (**B**) Number of DEGs on the 5th d (**C**) Number of DEGs on the 7th d (**D**) Venn diagram of DEGs. DEGs: |log2 FC| ≥ 0.5849, Q value ≤ 0.05.

The screened differentially expressed genes (DEGs) were analyzed as shown in Figure 4, compared to WT, the number of up-regulated and down-regulated DEGs in Δ*MrwetA* were 285 and 386 on the 3rd d (Figure 4A), 616 and 844 on the 5th d (Figure 4B), 690 and 1013 on the 7th d (Figure 4C), respectively. In addition, the number of common DEGs was 163 at all time points (Figure 4D). In summary, the number of DEGs increased along with cultivation, and the number of down-regulated DEGs was more than that of up-regulated DEGs at all time points. This trend indicates a potential stronger involvement of *MrwetA* in the up-regulation of gene expression.

#### 3.4.2. KEGG Pathway Enrichment Analysis of DEGs

**Figure 5:**
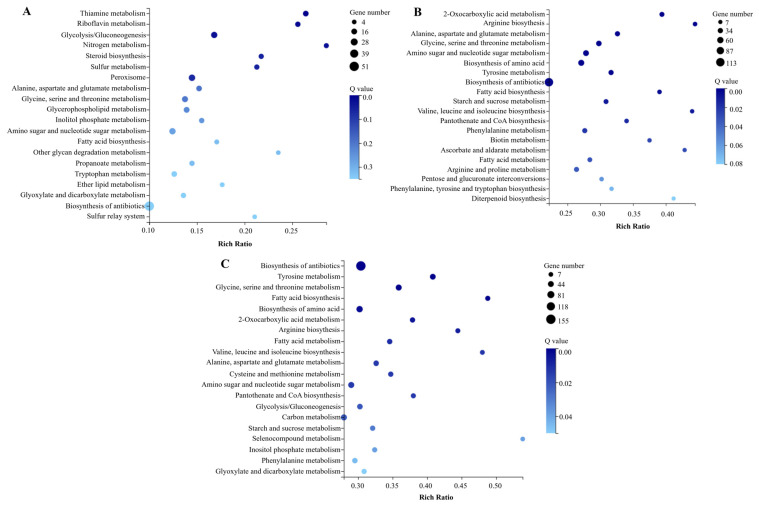
KEGG enrichment analysis of DEGs in WT vs. Δ*MrwetA*. The top 20 KEGG pathways enriched by DEGs on the 3rd d (**A**), 5th d (**B**), and 7th d (**C**). The size of the dot indicates the number of DEGs, and a smaller *p*-value indicates a more significant enrichment.

The functions of DEGs at three time points (3rd, 5th, and 7th d) were investigated from five categories by KEGG pathway, including cellular processes, environmental information processing, genetic information processing, metabolism, and organismal systems. Transport and catabolism, signal transduction, folding, sorting and degradation, carbohydrate metabolism, amino acid metabolism, lipid metabolism, and global and overview maps were the main KEGG subcategories in which DEGs were involved (Figure S4). On the 3rd d, key pathways enriched with DEGs included glycolysis/gluconeogenesis, riboflavin metabolism, and thiamine metabolism (Figure 5A). Subsequently, on the 5th and 7th d, major pathways enriched with DEGs were similar, including biosynthesis of antibiotics, biosynthesis of amino acids, amino sugar and nucleotide sugar metabolism, and glycine, serine and threonine metabolism (Figure 5B,C).

#### 3.4.3. Effects of *MrwetA* on Sexual Reproduction

**Figure 6:**
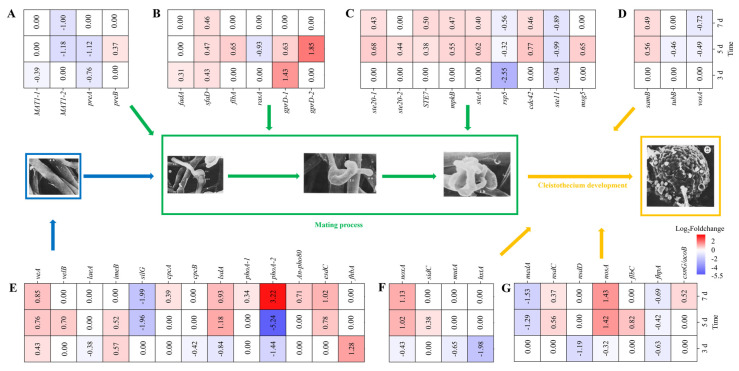
Differential expression of sexual reproduction-related genes in Δ*MrwetA* strain. (**A**) Mating-related genes; (**B**) G protein signaling pathway-related genes; (**C**) MAPK signaling pathway-related genes; (**D**) Fruiting body maturation-related genes; (**E**) Environmental signal reception-related genes; (**F**) Endogenous physiological processes-related genes; (**G**) Transcription factors and regulatory proteins-related genes. DEGs: |log2 FC| ≥ 0.5849, Q value ≤ 0.05; Positive number: upregulated; Negative number: downregulated; Blank: not DEGs. The microstructure of the cleistothecia development process was cited from Kolotila et al. [42].

As evidenced in Figure 2, the deletion of *MrwetA* inhibited the development of sexual structure cleistothecia. Both M7 and the model fungus *A. nidulans* belong to Aspergillaceae family and share a similar sexual reproductive process. Sexual reproduction is a complex biological process that requires the participation of many genes. To elucidate the molecular groundwork, BLASTP was used to identify homologues of sexual development-related genes, verified in *A. nidulans*, in the M7 genome (Table S3). Furthermore, the DEGs involved in M7 sexual reproduction were analyzed in six subgroups (Mating processes, signal transduction, perception of environmental signals, transcription factors, regulatory proteins, and fruiting body maturation) as shown in Figure 6.

Mating-type (*MAT*) genes and genes related to signal transduction pathways play a crucial role in regulating fungal sexual reproduction. In comparison with the M7, *MAT1-2* expression levels were down-regulated on the 5th and 7th d, and pheromone receptor *preA* (regulated by *MAT1-2*) expression levels were down-regulated on the 3rd and 5th d in the deletant (Figure 6A). G protein-coupled receptors (GPCRs), possessing seven transmembrane structures, are activated upon binding to pheromones and then bind to heterotrimeric G proteins. In G protein signaling pathway, the expression levels of *gprD-1* (encoding GPCR) were elevated on the 3rd and 5th d. In addition, the expression levels of *flbA* (regulator of G protein signaling) and *gprD-2* (encoding GPCR) were also elevated in Δ*MrwetA* on the 5th d, while the opposite was observed for *rasA* (GTPase) (Figure 6B). Moreover, in the MAPK (mitogen-activated protein kinase) cascade signaling pathway, the expression levels of *rsp5* (E3 ubiquitin-protein ligase) and *ste11* (mitogen-activated protein kinase) were down-regulated at different time points, while the other genes were up-regulated on the 5th and 7th d (Figure 6C). Among DEGs related to fruiting body maturation as shown in Figure 6D, only the expression level of *vosA* (trehalose production) decreased on the 5th and 7th d.

Light and nutrient factors in the environment serve as important signals for the fungal sexual reproduction process. As shown in Figure 6E, the expression levels of *veA* and *velB* related to light response increased on the 5th and 7th d, while the opposite was observed for *silG*. Changes in the expression levels of *lsdA*, *phoA*, *An-pho80*, and *fhbA* related to nutrient perception varied across time points. In addition, the expression levels of *esdC*, which regulates early sexual development, increased on the 5th and 7th d. On the other hand, DEGs related to various physiological processes (Figure 6F) demonstrated decreased expression levels of *noxA* (NADPH oxidase), *mutA* (α-1,3 glucanase/mutanase), and *hxtA* (high-affinity hexose transporter) on the 3rd d compared with WT. Moreover, the expression of *noxA* was increased on the 5th and 7th d.

Transcription factors and genes related to fruiting body maturation are indispensable from the mating process to the maturation of cleistothecia. In transcription factors, compared with WT, the expression levels of *nsdD* and *fhpA* decreased on the 3rd d; On the 5th d, the expression levels of *nsdC*, *nosA*, and *flbC* increased, while *medA* decreased; on the 7th d, both *nosA* expression levels increased, in contrast to *fhpA* (Figure 6G).

In summary, *MrwetA* regulated various genes associated with the sexual reproduction of M7, consistent with the findings presented in Figure 2.

#### 3.4.4. Effects of MrwetA on Meiosis

**Figure 7:**
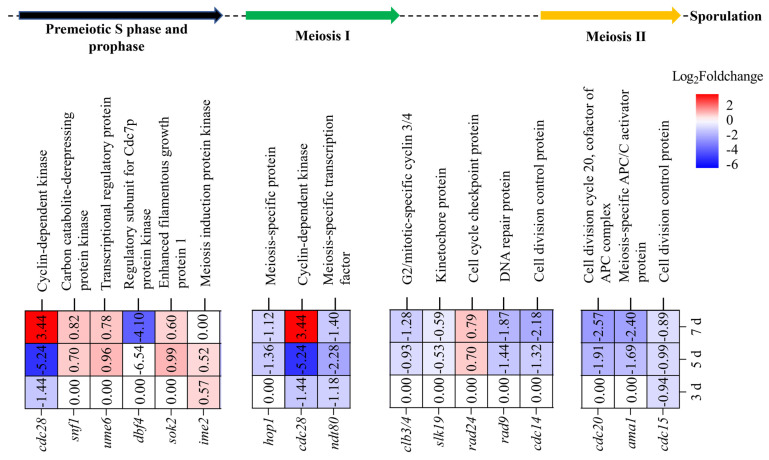
Differential expression of meiosis-related genes in Δ*MrwetA* strain. DEGs: |log2 FC| ≥ 0.5849, Q value ≤ 0.05; Positive number: upregulated; Negative number: downregulated; Blank: not DEGs.

During sexual reproduction in fungi, the formation of haploid spores requires meiosis. As depicted in Figure 7, five DEGs (*cdc28*, *snf1*, *ume6*, *dbf4*, and *sok2*) were detected during the Premeiotic S phase and prophase, three DEGs (*hop1*, *cdc28*, and *ndt80*) during meiosis I, and three DEGs (*cdc20*, *ama1*, and *cdc15*) during meiosis II. Additionally, five DEGs (*clb3/4*, *slk19*, *rad24*, *rad9*, and *cdc14*) were detected between meiosis I and meiosis II. Compared with WT, the expression levels of *cdc28*, *ndt80*, and *cdc15* were decreased on the 3rd d. Furthermore, the number of DEGs on the 5th and 7th d significantly exceeded that of the 3rd d, with a consistent expression trend observed at both time points, predominantly characterized by decreased expression levels among the majority of DEGs. These changes in DEGs involved in meiosis align with the morphology and yield changes in cleistothecia and ascospores.

#### 3.4.5. Effects of MrwetA on Secondary Metabolites

**Figure 8:**
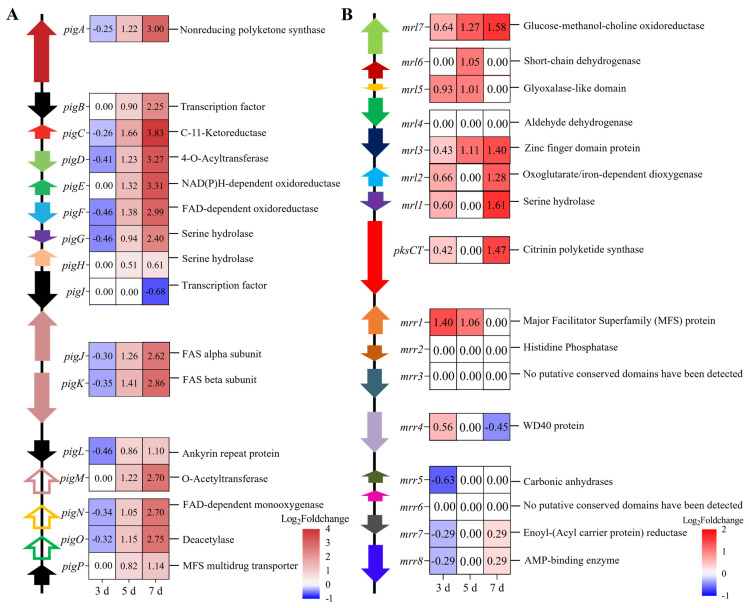
Differential expression of the biosynthesis of MPs (**A**) and CIT (**B**) related genes in Δ*MrwetA* strain. DEGs: |log2 FC| ≥ 0.5849, Q value ≤ 0.05; Positive number: upregulated; Negative number: downregulated; Blank: not DEGs.

MPs and CIT are the main secondary metabolites in M7 and deletion of *MrwetA* disturbed the yield of these products. To elucidate the underlying mechanism, the genes involved in the biosynthesis of MPs and CIT [11,12,13] were analyzed (Figure 8). Compared with WT, there was no significant difference in the expression level of genes related to MPs biosynthesis on the 3rd d in Δ*MrwetA* (Figure 8A). However, the expression levels of *MrPigA*-*G* and *MrPigJ*-*P* were significantly higher on the 5th and 7th d, while the expression level of *MrPigI* decreased only on the 7th d. 

According to He and Cox. [11], key genes in the CIT major biosynthetic pathway include *pksCT*, *mrl1*, *mrl2*, *mrl4*, *mrl6*, and *mrl7*. As depicted in Figure 8B, the expression levels of *mrl1*, *mrl2*, *mrl5*, and *mrl7* increased on the 3rd d, *mrl3* and *mrl5*-*7* on the 5th d, *pksCT*, *mrl1*-*3*, and *mrl7* on the 7th d in Δ*MrwetA* compared with WT. These changes in MPs and CIT biosynthesis-related genes were consistent with the increased yield of MPs and CIT, indicating that *MrwetA* regulated the production of MPs and CIT through the biosynthesis pathway.

## 4. Discussion and Conclusions

Based on the previous findings [20], deletion of *MrwetA* hindered sexual development and affected the yield of secondary metabolites, while complementation of *MrwetA* totally restored these observed disorders, verifying its role in sexual development and biosynthesis of secondary metabolites. On the other hand, *MrwetA* deletants and overexpressed mutants both exhibited defects in asexual development [20]. This pattern of consistent phenotypes resulting from both deletion and overexpression is also observed in genes in other fungi, where the strict expression levels of these genes are crucial either for their own functionality [43,44] or for their involvement in the formation of protein complexes [45] essential for an organism to form protein complexes for organisms. Although this study confirmed the important role of M7, especially for the cleistothecia, further investigation is needed to determine whether it works through complex or solely. Furthermore, in this study, 1703 DEGs (|log2 FC| ≥ 0.5849, Q value ≤ 0.05) were detected on the 7th d, accounting for 22.2% of all genes (9% up-regulated and 13.2% down-regulated) of all genes. However, the RNA-seq data showed that after *wetA* deletion, DEGs (|log2 FC| ≥ 1, Q value ≤ 0.05) accounted for 52% (28% up-regulated and 24% down-regulated), 57% (30% up-regulated and 27% down-regulated), 43% (21.6% up-regulated and 21.4% down-regulated) of all genes in *A. nidulans*, *A. fumigatus*, and *A. flavus*, respectively [34]. The percentage of *wetA*-regulated genes in M7 was significantly lower than those in the aforementioned species. Additionally, a larger proportion of down-regulated genes was observed, suggesting a unique regulatory network of *wetA* in M7.

*wetA* has been reported to regulate asexual reproduction in various filamentous fungi such as *A. nidulans*, *A. fumigatus*, *F. graminearum*, and *B. bassiana* [25,30,46,47]. However, disruption of it in M7 hindered sexual reproduction and showed no effect on asexual reproduction [20]. The underlying mechanism remains elusive, because of the complexity of sexual reproduction involving numerous genes. Referred to Dyer and O’Gorman [17], the genes related to sexual reproduction in M7 also can be categorized into six groups (Mating processes, signal transduction, perception of environmental signals, transcription factors, regulatory proteins, and fruiting body maturation).

*MAT* genes play a pivotal role in fungal sexual reproduction, orchestrating the expression of pheromone-related genes and GPCRs, which are pivotal for downstream signaling cascades, including the MAPK pathway, essential for sexual development [48,49,50]. In this study, there have been alterations in the expression levels of *MAT1-2*, *gprD*, *rasA*, and *ste11*, which are involved in MAT processes, GPCRs, and the MAPK pathway, respectively. Furthermore, environmental signal reception, transcription factors, endogenous physiological processes and genes related to fruiting body maturation also participate in the process of sexual reproduction. In our study, expression changes in genes related to these processes were identified, suggesting their involvement in sexual reproduction regulation. For instance, changes in the expression of *vosA* and *noxA* genes imply alterations in trehalose production and redox reactions, respectively, influencing cleistothecia development.

Meiosis is another necessary process for the sexual reproduction of haploid spores in filamentous fungi. In budding yeast, specific transcription factors such as *ime1* initiate the meiosis process, subsequently regulating the expression of genes including *ime2* and *cdc28* to promote meiosis [51,52]. Based on RNA-Seq data, the expression levels of *cdc28*, *ndt80*, and *cdc15* were significantly decreased on the 3rd d, while the number of down-regulated genes increased on the 5th and 7th d. This suggests a delayed onset of meiosis-related gene expression in Δ*MrwetA*, potentially contributing to the observed reduction in cleistothecia and ascospores.

In addition to interfering with sexual reproduction, *MrwetA* also affects the biosynthesis of MPs and CIT. The biosynthesis of MPs and CIT is regulated by various genes including *MrpigA-P* and *pksCT*, *mrl1-7* [12,13,53,54,55,56,57]. In this study, the yield of MPs in Δ*MrwetA* was elevated compared to WT on the 7th d, accompanied by increased expression of *MrpigA* (polyketide synthase *pksPT*), *MrpigB* (transcription factor), *MrpigC* (C-11-ketoreductase), *MrpigD* (4-O-acyltransferase), *MrpigE* (NAD(P)H-dependent oxidoreductase), *MrpigF* (FAD-dependent oxidoreductase), *MrpigG* (serine hydrolase), *MrpigJ* (FAS alpha subunit), *MrpigK* (FAS beta subunit), *MrpigL* (ankyrin repeat protein), *MrpigM* (O-acetyltransferase), *MrpigN* (FAD-dependent monooxygenase), *MrpigO* (deacetylase), and *MrpigP* (MFS multidrug transporter) on the 5th and 7th d (Figure 8A). According to Li et al. [13] and Chen et al. [12], *MrpigA*, *MrpigC-G*, *MrpigJ-K*, *MrpigM*, and *MrpigO* have been confirmed to be directly involved in the biosynthetic pathway of MPs. Similarly, CIT production increased in Δ*MrwetA*, correlating with the upregulated genes including *pksCT* (polyketide synthase), *mrl1* (serine hydrolase), *mrl2* (oxoglutarate/iron-dependent dioxygenase), *mrl4* (aldehyde dehydrogenase), *mrl6* (short-chain dehydrogenase), and *mrl7* (glucose-methanol-choline oxidoreductase). The above results indicate that *MrwetA* can change MPs and CIT yield by regulating the expression of the biosynthesis gene.

In summary, our findings underscore the role of *MrwetA* in the sexual development and secondary metabolism of M7. By affecting the expression of genes involved in sexual reproduction, meiosis, and secondary metabolite biosynthesis, *MrwetA* contributes to the regulatory network governing these processes. These insights advanced our understanding of reproduction and metabolism in M7, while also broadening our knowledge of *wetA* function and sexual reproduction regulation in filamentous fungi.

## Figures and Tables

**Table 1:** Strains used in this study.

Strain	Parent	Genotype	Source
M7	M7	Wild-type	[35]
Δ*MrwetA*	M7	Δ*MrwetA*::*hph*	[20]
Δ*MrwetA*::*MrwetA*	Δ*MrwetA*	Δ*MrwetA*::*MrwetA*-*neo*	This study

## Data Availability

Data are contained within the article and Supplementary Materials.

## Supplementary Materials

The following supporting information can be downloaded at: https://www.mdpi.com/article/10.3390/jof10050338/s1, Figure S1. Schematic diagram of constructing transformation plasmid pCCMrwetA. Figure S2. Verification of PCR products and vector on *MrwetA* complementation cassette. Figure S3. PCR verification of *MrwetA* complementation strain. Figure S4. KEGG Pathway classification of DGEs. Table S1. Primers used in the construction of *MrwetA* complementation strain. Table S2. Primer combinations and expected sizes for validation of Δ*MrwetA*::*MrwetA* strains. Table S3. Homologues of sexual reproduction related genes of *Aspergillus nidulans* in *Monascus ruber* M7.
