# Supplementary material for: Effects of MrwetA on Sexual Reproduction and Secondary Metabolism of Monascus ruber M7 Based on Transcriptome Analysis
Source: J Fungi (Basel). 2024 May 8;10(5):338. doi: 10.3390/jof10050338 (PMC11122622; doi:10.3390/jof10050338)

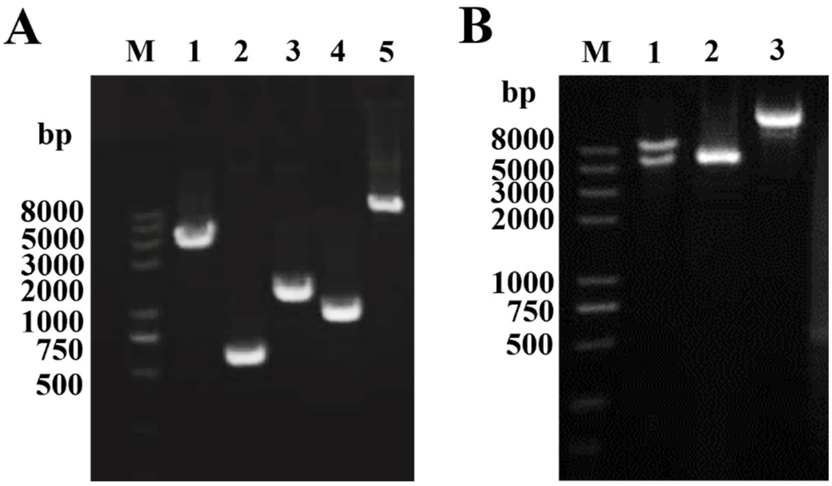

Figure S2. Verification of PCR products and vector on *MrwetA* complementation cassette. A: M: marker; Lane 1: 5'UTR-*MrwetA*; Lane 2: *TtrpC*; Lane 3: *neo*; Lane 4: 3'UTR; Lane 5: *MrwetA* complementation cassette. B: M: marker; Lane 1: digested products; Lane 2: *MrwetA* complementation cassette; Lane 3: pCCMrwetA

The original images are as follows:

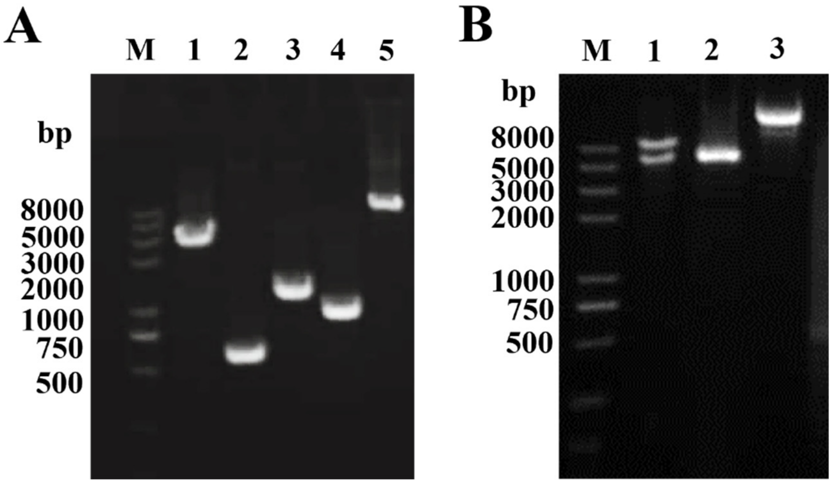

Supplement: Supplementary file 1 [file jof-10-00338-s001.zip › Figure S2. Verification of PCR products and vector on MrwetA complementation cassette.pdf]
