# Supplementary material for: Effects of MrwetA on Sexual Reproduction and Secondary Metabolism of Monascus ruber M7 Based on Transcriptome Analysis
Source: J Fungi (Basel). 2024 May 8;10(5):338. doi: 10.3390/jof10050338 (PMC11122622; doi:10.3390/jof10050338)

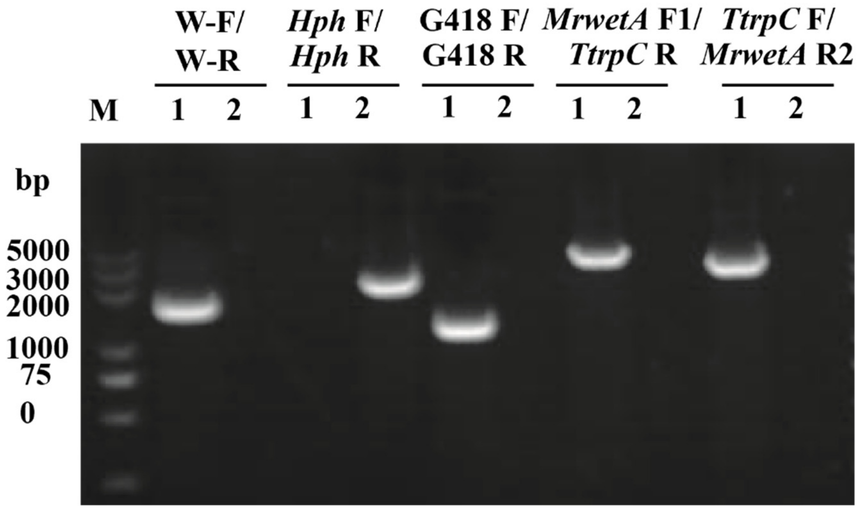

Figure S3. PCR verification of *MrwetA* complementation strain. M: marker; Lane 1 and 2: The genomes of the Δ*MrwetA*::*MrwetA* and Δ*MrwetA* strains were used as templates.

The original images are as follows:

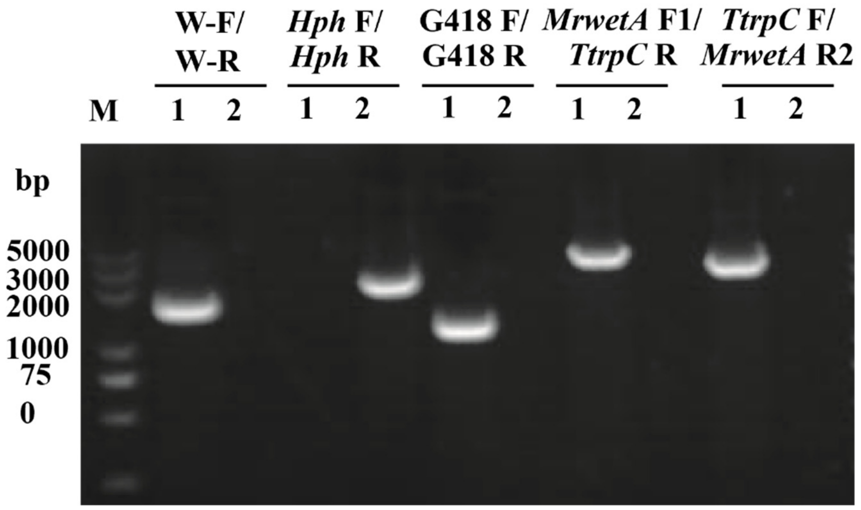

Supplement: Supplementary file 1 [file jof-10-00338-s001.zip › Figure S3. PCR verification of MrwetA complementation strain.pdf]
