# Supplementary material for: Effects of MrwetA on Sexual Reproduction and Secondary Metabolism of Monascus ruber M7 Based on Transcriptome Analysis
Source: J Fungi (Basel). 2024 May 8;10(5):338. doi: 10.3390/jof10050338 (PMC11122622; doi:10.3390/jof10050338)
Supplement: Supplementary file 1 [file jof-10-00338-s001.zip › Figure S4. KEGG Pathway classification of DGEs..pdf]

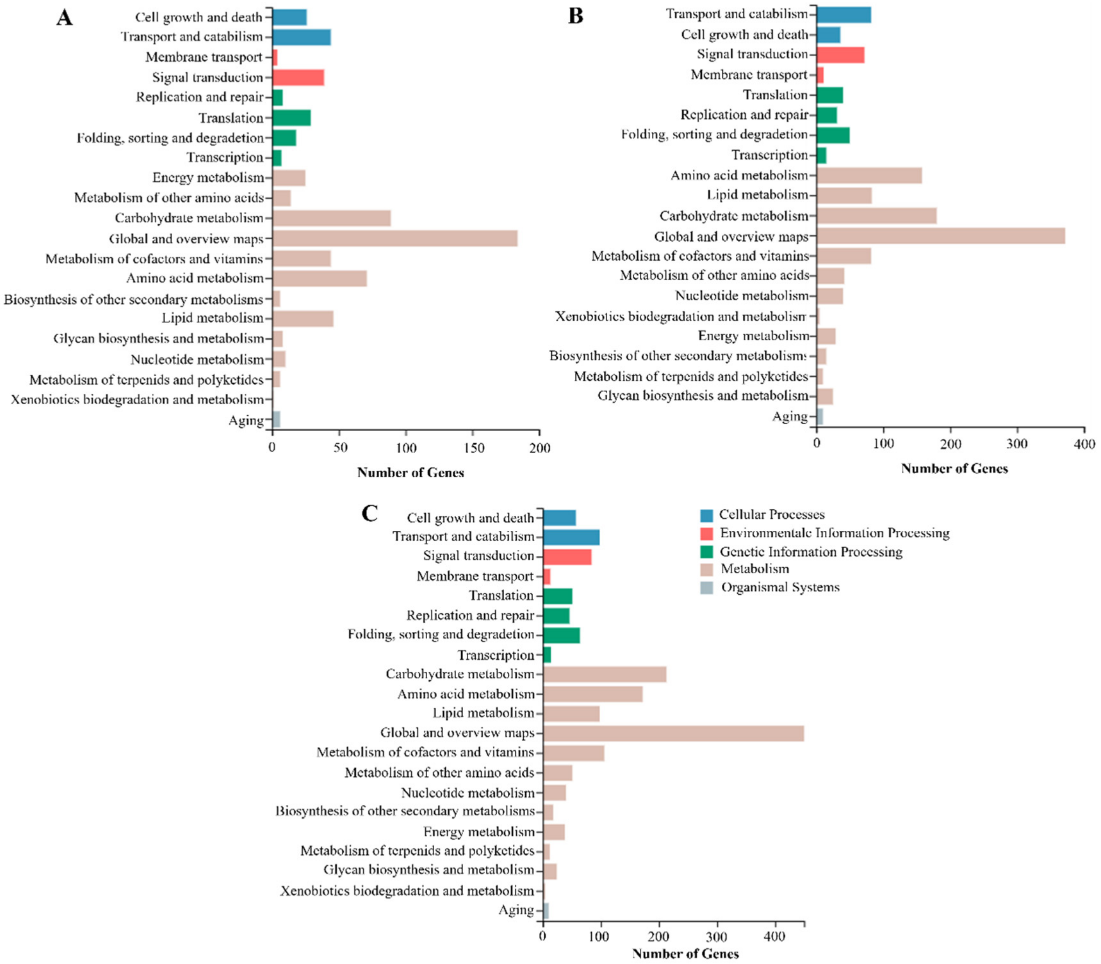

Figure S4. KEGG Pathway classification of DGES. A, B and C: KEGG Pathway classification of DEGs on the 3rd d (A), 5th d (B) and 7th d (C)
