# Supplementary figures and images for: Effects of MrwetA on Sexual Reproduction and Secondary Metabolism of Monascus ruber M7 Based on Transcriptome Analysis
Source: J Fungi (Basel). 2024 May 8;10(5):338. doi: 10.3390/jof10050338 (PMC11122622; doi:10.3390/jof10050338)

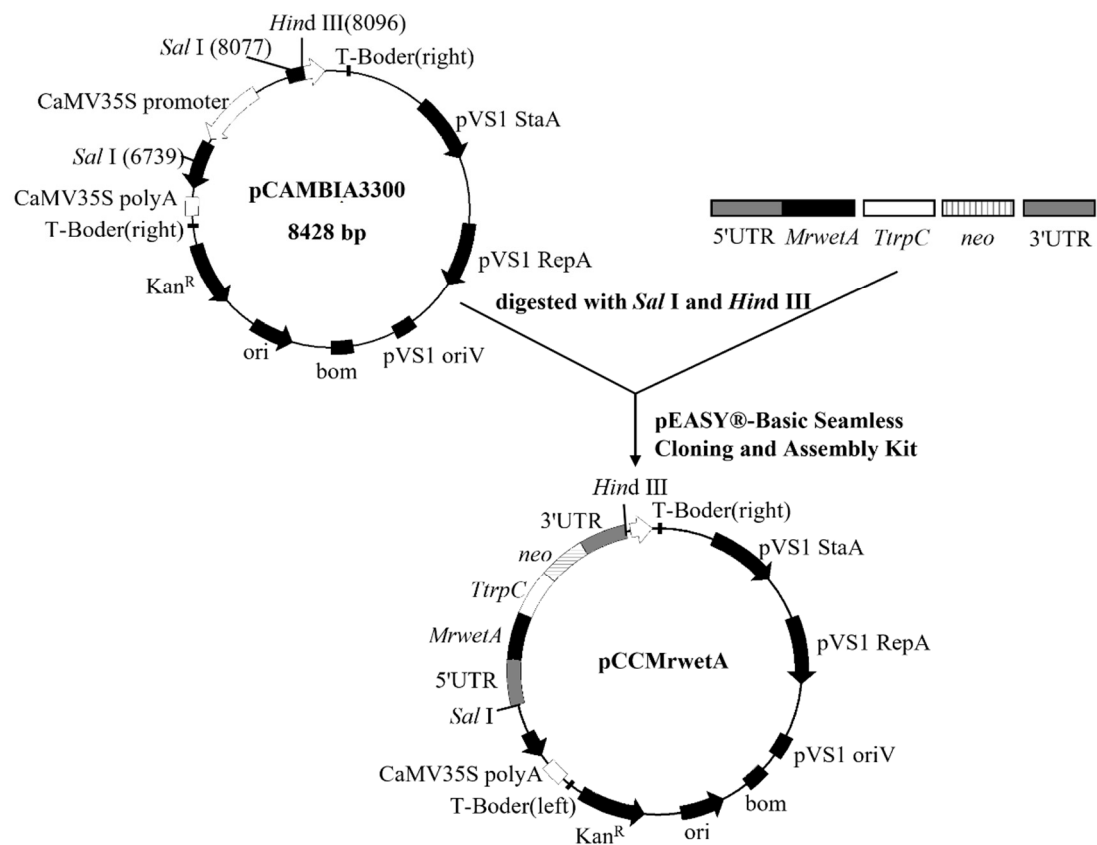

**Figure S1.** Schematic diagram of constructing transformation plasmid pCCMrwetA

Supplement: Supplementary file 1 [file jof-10-00338-s001.zip › Figure S1. Schematic diagram of constructing transformation plasmid pCCMrwetA.pdf]
